# Shaping the founders: naïve CD4 T cell heterogeneity in people with HIV-1 or HIV-2

**DOI:** 10.3389/fimmu.2026.1810186

**Published:** 2026-05-11

**Authors:** Robert Badura, Nicole C. Martins, Guilherme B. Farias, Rita Tendeiro, Russell B. Foxall, Diana F. Santos, André M. C. Gomes, Rita T. Marques, Beatriz Moleirinho, Ana C. Godinho-Santos, Emília Valadas, Perpétua Gomes, Afonso R. M. Almeida, Ana E. Sousa

**Affiliations:** 1 GIMM - Gulbenkian Institute for Molecular Medicine, Lisbon, Portugal; 2 Faculdade de Medicina, Universidade de Lisboa, Lisboa, Portugal; 3 Hospital de Santa Maria, Unidade Local de Saúde Santa Maria, Lisboa, Portugal; 4 Egas Moniz Center for Interdisciplinary Research (CiiEM), Egas Moniz School of Health & Science, Almada, Portugal; 5 Laboratório de Biologia Molecular, SPC, Unidade Local de Saúde Lisboa Ocidental- Hospital Egas Moniz, Lisboa, Portugal

**Keywords:** acute HIV-1 infection, CD31, elite controllers, HIV/AIDS, HIV-2, IL-7, memory stem-cells, naïve CD4 T cells

## Abstract

Naïve CD4 T cells harbour functional subsets that influence the quality of immune reconstitution and the persistent viral reservoirs in people with HIV (PWH) under antiretroviral therapy (ART). Here, we profiled the circulating naïve CD4 T cells from PWH under effective ART with distinct past histories of viral burden, namely starting treatment early in acute or late in chronic stage HIV-1 infection, and people with HIV-2 (PWH2), who feature low to undetectable viremia before ART and slow disease progression. Spectral flow cytometry enabled us to capture the heterogeneity of this overlooked T cell compartment. Early-treated PWH1 maintained a naïve CD4 T cell profile comparable to that of age-matched seronegative individuals. Late-treated PWH1 and PWH2 showed distinct imbalances in conventional and regulatory subpopulations, yet both exhibited a relative expansion of CD31-expressing naïve cells, consistent with an IL-7-mediated homeostatic response. The ability of purified naïve CD4 T cells to respond to IL-7 was evaluated in additional cohorts of untreated PWH, revealing reduced proliferation and increased p21 transcription in PWH1 elite controllers. Additionally, a significant decline in proviral DNA was found in PWH2, suggesting an impact of IL-7 on HIV-2-infected naïve CD4 T cells that was not observed in the case of HIV-1 infection. Unravelling the homeostatic pathways and functional implications of naïve CD4 T cell heterogeneity will help clarify viral reservoir dynamics and inflammation in PWH and define strategies to prevent immune senescence.

## 1 Introduction

The naïve CD4 T cell subset is now recognised to comprise several functional populations with distinct roles in ensuring immune competence throughout life (1–3). The naïve compartment is continuously replenished by the thymic output of cells called “recent thymic emigrants” (RTEs), known to express high levels of CD31 and CD38 (2, 4, 5) and to have unique properties for responding to IL-7 and proliferating (6). CD31 is thought to be lost upon naïve CD4 T cell proliferation in the periphery, and the CD31^neg^ naïve subset is mainly maintained by T cell receptor (TCR) induced proliferation mediated by low-affinity self-peptide interactions (4). A significant population of thymically-committed regulatory T cells exists expressing lower levels of FOXP3 and CD25 than their memory counterparts, which rapidly acquire a suppressive phenotype upon stimulation (7). There are also stem cell memory T cells (TSCM), a small population of antigen-experienced cells with stem properties, namely asymmetric division with self-maintenance in parallel with rapid differentiation into the memory pool, defined by the expression of CXCR3 and/or CD95, high levels of CD122, the IL-2β receptor, and the ability to produce IFNγ (8). Moreover, there are increasing reports of memory CD4 T cells with a naïve-like phenotype induced upon infections (9).

HIV mainly infects memory, activated and proliferating T cells (10, 11). Notwithstanding this, naïve T cells were shown to be infected *in vivo* (12–14), and *in vitro* (15), particularly upon overcoming the host-restriction factor SAMHD1 (15). The vpx protein, present in HIV-2 and SIV but not in HIV-1, can promote SAMHD1 ubiquitination and, in this way and possibly others, facilitate HIV infection of quiescent cells (16, 17). In addition, both HIV-1 and HIV-2 infect thymocytes (18, 19), although people with HIV-2 (PWH2) were shown to better preserve thymic output, as assessed by TCR excision circles (TRECs), than PWH1 (20, 21). Importantly, CD4 TSCM have been shown to harbour a significant latent HIV reservoir and to be the main determinants of viral reservoir stability upon ART in PWH1 (12, 22, 23).

It is also worth emphasising that both PWH1 and PWH2 feature persistent immune activation in correlation with the degree of CD4 T cell depletion (24), which may also impact the naïve compartment (25) and alter its homeostasis by promoting a continuous pressure for memory differentiation and impairing the naïve homeostatic niches in the lymph nodes (26).

Here, we aim to investigate the heterogeneity of naïve CD4 T cells under suppressive ART in PWH1 with distinct past histories of viral burden, namely starting treatment early in acute HIV-1 infection or in advanced chronic stage, and in PWH2. PWH2 were included because they are known to feature low to undetectable viremia even before ART and to have a very slow rate of disease progression, configuring HIV-2 infection as a naturally-occurring attenuated form of HIV/AIDS (24).

## 2 Materials and methods

### 2.1 Human samples and ethics statement

Cross-sectional study of *ex vivo* naïve CD4 T cells from 60 PWH, including: a rare group of 16 PWH1 who started ART in very early Fiebig stages, PWH1(AI), a previously described cohort (27, 28); 19 PWH1(C) who initiated ART during the chronic stage; and 18 PWH2 with undetectable plasma viral load; together with 21 age-matched seronegative controls (**Table 1**). In addition to these virally suppressed PWH cohorts, viraemic PWH were enrolled in parallel, specifically 3 acute HIV-1 seroconverting individuals (PWH1_Untreated_AI), 3 PWH1 at diagnosis in the chronic stage (PWH1_Untreated_C), and 1 PWH2 with detectable viremia (PWH2_Viraemic). Another group of 34 subjects was selected for the IL-7 response study, which was conducted before the universal use of ART and designed to include only ART-naïve PWH without major CD4 T cell depletion (more than 300 CD4 T cells/µl), namely: 5 rare cases (< 0.1%) of untreated HIV-1+ individuals with undetectable viremia, formally defined as elite controllers (EC) (29, 30); PWH1 non-controllers (NC) featuring the expected high viremia; and typical untreated PWH2 with low to undetectable plasma viral load (24, 31); along with age-matched seronegative controls (**Table 2**). All PWH were under follow-up at the Department for Infectious Diseases, Unidade Local de Saúde Santa Maria, Lisboa, Portugal. Individuals with cancer, autoimmune disease, and ongoing opportunistic infections were excluded. Blood lymphocyte counts, viremia and inflammatory markers were collected from the clinical registry. HIV-2 viremia was quantified at Hospital Egas Moniz, Unidade Local de Saúde Lisboa Ocidental, Lisboa, Portugal, by Perpétua Gomes (32). The study was approved by the Ethics Committee of the Centro Académico de Medicina de Lisboa (CAML) and conducted after obtaining signed informed consent from all participants.

**Table 1:** Epidemiological data of aviraemic PWH and seronegative cohorts.

Parameter	Seronegative	PWH1(AI)	PWH1(C)	PWH2
N [male/female]	21 [11/10]	16 [14/2]^§§§^	19 [9/10]	18 [3/15]
Caucasian/other	21/0	12/4	14/5	4/14
Age, years	51 ± 2(33-65)	45 ± 3(27-75)^§§^	54 ± 3(25-73)	60 ± 3(34-76)
Time on ART, years	NA	6.2 ± 0.6(3-10)^a^	11.5 ± 1.7(1-24)	6.7 ± 1.6(1-20)^b^
Lymphocytes/µl	2366 ± 131(1480-4259)	1712 ± 113(978-2778)^**^	2067 ± 122(1092-2895)	1723 ± 131(772-3019)^**^
% CD4 T cells	48.3 ± 1.8(36.7-63.5)	38.6 ± 1.5(28.4-51.7)^##^	24.9 ± 1.6 (12.5-41.2)^***^	32.6 ± 2.6(16.0-56.8)^***^
CD4 T cells/µl	1154 ± 93(642-2703)	670± 57(277-1091)^**^	509 ± 40(224-843)^***^	571 ± 61(164-1115)^***^
% CD8 T cells	22.8 ± 1.3(8.3-31.9)	27.8 ± 1.9(12.4-40.8)^###^	46.9 ± 3.0(28.3-81.6)^***, §^	31.9 ± 2.3(11.6-48.7)^*^
CD8 T cells/µl	546± ± 45(174-900)	463± 36(221-756) ^###^	980 ± 100(445-2363)^**, §§^	550 ± 70(278-1470)
CD4/CD8	2.39 ± 0.28(1.26-6.91)	1.56 ± 0.18(0.76-3.09)^###^	0.59 ± 0.06(0.15-1.09)^***, §^	1.20± 0.16(0.34-2.80)^**^
% naïve CD4 T cells	39.8 ± 2.8(14.9-68.4)	36.4 ± 2.2(14.7-50.5)	30.0 ± 3.9(7.9-57.8)	31.8 ± 4.1(9.7-28.6)
Naïve CD4 T cells/µl	486 ± 69(140-1592)	249 ± 30(77-551)	147 ± 25(34-488)^***^	201 ± 39(16-652)^***^
Cell-associated Viral DNA, cps/10^6^PBMC^c^	NA	541 ± 197(88-3028) ^§§c^	365 ± 68(56-1018) ^§§^	149± 40(18-402)^c^
IL-6, pg/mL	NA	3.58 ± 1.21(1.50-21.50)^§^	5.87 ± 2.28(1.60-46.10)	4.92 ± 1.12(1.50-22.00)
D-dimers, μg/mL	NA	0.36 ± 0.05 (0.20-1.12)	0.35 ± 0.04(0.19-0.76)^§^	0.55 ± 0.13(0.15-2.15)^d^
β2m, mg/L	NA	1.90 ± 0.11(1.03-2.86)	2.53 ± 0.33(1.31-7.36)	2.90 ± 0.68(1.32-13.47)

Data are Mean ± SEM with limits in brackets, unless otherwise indicated. NA, not applicable. Data were compared using Kruskal-Wallis test and Dunn’s test. Statistical differences: between a given PWH cohort and seronegative controls: *, *p* < 0.05; **, *p* < 0.01; ***, *p* < 0.001; between PWH1(C) and PWH2 cohort: §, *p* < 0.05; §§, *p* < 0.01; §§§ *p* < 0. 001; and between PWH1(AI) and PWH1(C): # *p* < 0.05; ## *p* < 0.01; ### *p* < 0.001. Reference values: IL-6 <1.5 pg/dL; D-dimers: 0.0-0.5 µg/dL, β2m: 0.8-3.0 mg/dL. ^a^ Fiebig stage at ART initiation: 7 in Fiebig 2; 5 in Fiebig 3 and 4 in Fiebig 4. ^b^ n=15 on ART; 3 PWH2 remained untreated; all with undetectable plasma viral load. ^c^ undetectable in 1 PWH1(AI) and 3 PWH2, which were not included. ^d^ analysis in 16 PWH2.

**Table 2:** Epidemiological data of untreated PWH and seronegative cohorts from the IL-7 study.

Parameter	Seronegative	PWH1-EC	PWH1- NC	PWH2
N (male/female)	15 (6/9)	5 (3/2)	12 (7/5)	12 (3/9)
Age, years	43 ± 3(26-63)	48 ± 6(36-67)	43 ± 4(25-63)	51 ± 3(33-63)
Caucasian/other	15/0	3/2	9/3	8/4
% CD4 T cells	46 ± 3(26-61)	41 ± 4(26-51)^#^	25 ± 2(18-50)^***^	35 ± 2(27-46)^*/###^
CD4 T cells/µl	1091 ± 71(860-1649)	826 ± 192(531-1575)	569 ± 52(300-939)^***^	829 ± 101(418-1464)^*^
% Naïve CD4 T cells^a^	20 ± 2(7-35)	20 ± 3(8-25)	12 ± 2(6-28)^*^	15 ± 2(6-28)
Naïve CD4 T cells/µl	449 ± 56(167-798)	394 ± 125(174-872)	260 ± 33(111-518)^*^	350 ± 47(118-684)
% HLA-DR^+^CD38^+^ in CD4	1 ± 0.2(0.5-2)	2 ± 0.1(2-3)^**^	5 ± 1(1-11)^***^	3 ± 0.5(1-7)*
% HLA-DR^+^CD38^+^ in CD8	5 ± 1(0.7-13)	17 ± 3(7-24)^**^	32 ± 4(13-54)^***^	13 ± 1(6-20)^**/##^
Viremia, RNA cps/ml	NA	<40	3x10^4^ ± 10^4^(295-7.3x10^4^)	65 ± 17(<40-211)^###^

Data are Mean ± SEM with limits in brackets, unless otherwise indicated. EC, elite controllers; NC, non-controllers; NA, not applicable. Data were compared using Mann-Whitney test. Statistical differences between a given PWH cohort and seronegative controls: *, *p* < 0.05; **, *p* < 0.01; ***, *p* < 0.001. Statistical differences between PWH1-NC and PWH2-EC or PWH2 cohort: #, *p* < 0.05; ##, *p* < 0.01; ### *p* < 0.001. ^a^ Defined as the percentage of CD4^+^CD45RA^+^ cells, in a lymphocyte gate of whole PBMC, according to forward and side scatter characteristics.

### 2.2 Flow cytometry - *ex vivo* naïve CD4 profile

Peripheral blood mononuclear cells (PBMCs) were isolated from freshly collected heparinised blood by density gradient centrifugation (Ficoll-Paque™ PLUS, Cytiva) and immediately stained with the monoclonal antibodies listed in **Supplementary Table 1**. Briefly, 2x10^6^ cells were incubated with the antibodies against surface antigens in the presence of FcR Blocking Reagent (Miltenyi Biotec) for 25min at room temperature; after washing, cells were fixed and permeabilized (Fixation/Permeabilization Buffer, eBioscience, 30min at 4°C) and incubated with the antibodies against intracellular markers for 30min at 4°C; after a final wash, cells were resuspended in PBS/BSA/Az and immediately acquired and compensated on a spectral flow cytometer (Aurora-3L-16V-14B-8R, Cytek®) using the SpectroFlo® software (v3.0.1). Data were manually analysed using FCS Express software (v7.14.0020). Lymphocytes were gated based on forward and side scatter (FSC/SSC) parameters, and after doublet exclusion, naïve CD4 T cells were defined as CD45+CD3+CD4+CCR7+CD45ROneg, as illustrated in the **Supplementary Figure 1A**. FCS files of gated naïve CD4 T cells were imported into R (v4.3.2; R Foundation for Statistical Computing) as flowSets using the flowCore package (v2.14.1). An arcsinh transformation was applied to all samples using flowVS (v1.34.0). Upon automated quality control (flowAI, v1.32.0), and normalisation (daNorm from the flowStats package, v4.14.1), down-sampling to the minimum event count across samples (11,307 naïve CD4 T cells per sample) was done, and UMAP was used for dimensionality reduction (uwot, v0.1.16), based on the expression values of all markers excluding those used for prior gating and SAMHD-1, due to batch effects in some samples. Clustering was performed using FlowSOM (CATALYST v1.26.0), and cluster structure visualised using clustree (v0.5.1). The number of clusters was determined based on tree stabilisation, with manual merging of clusters exhibiting phenotypic similarity, and exclusion of low-frequency or participant-specific clusters (those separating early in the tree), as shown in **Supplementary Figures 1B, C**.

### 2.3 Naïve CD4 T cell culture

PBMCs, isolated from heparinised blood immediately after collection (Ficoll-Paque™ PLUS, Cytiva), were purified into naïve CD4 T cells by immunomagnetic separation according to the manufacturer’s instructions. Two strategies were employed: direct negative selection using the Human Naïve CD4 T cell Enrichment Kit (StemCell Technologies), or a two-step protocol involving initial negative selection of total CD4 T cells (Human CD4 T cell Enrichment Kit, StemCell Technologies) followed by depletion of memory T cells using CD45RO microBeads (Miltenyi Biotec). The purity of sorted naïve CD4 T cells assessed by flow cytometry was 95 ± 1% for seronegative controls, 92 ± 1% for PWH1-NC, 94 ± 1% for PWH2, and 96 ± 1% for PWH1-EC. Purified naïve CD4 T cells were cultured at a density of 1x10^6^ cells/mL, in 96-well round-bottom plates, with RPMI 1640 medium supplemented with 100 U/mL penicillin, 100 µg/mL streptomycin, 2 mM L-glutamine (all from Gibco-Invitrogen), and 10% human AB serum (Sigma-Aldrich). Cells were stimulated with either IL-7 (10 ng/mL) or IL-2 (20 U/mL) and maintained at 37 °C in a humidified chamber with 5% CO_2_. On day 3, cultures were replenished with fresh medium supplemented with the appropriate cytokine. After 7 days, cells were harvested for flow cytometry analysis and DNA/RNA extraction.

### 2.4 Flow cytometry analysis of stimulated naïve CD4 T cells

Surface and intracellular staining were performed both *ex vivo* and after 7-day culture, as previously described (33), using the panel of antibodies detailed in **Supplementary Table 2**. Apoptotic cells were identified using Annexin V staining with the Annexin V-FITC Apoptosis Detection Kit I (BD Biosciences), according to the manufacturer’s instructions, with samples acquired within one hour of completing the staining protocol. Data acquisition was performed using either a FACSCanto™ or FACSCAlibur™ flow cytometer (BD Biosciences). A minimum of 50,000 events (FACSCalibur™) or 100,000 events (FACSCanto™) were acquired per sample, and analysis was performed using FlowJo software (BD Biosciences).

### 2.5 RNA isolation and qRT-PCR

Pellets containing a minimum of 250,000 naïve CD4 T cells were harvested immediately after isolation and following 7-day culture with either IL-2 or IL-7. Cells were lysed in RLT Buffer (Qiagen), and total RNA and DNA were extracted using the AllPrep DNA/RNA Micro kit (also from Qiagen), according to the manufacturer’s protocol. Total RNA was eluted in RNase-free water and quantified using a Nanodrop ND1000 spectrophotometer (Saveen Biotech ApS). For cDNA synthesis, 50 ng of total RNA was reverse transcribed using Superscript III Reverse Transcriptase (Life Technologies), following the manufacturer’s instructions. Quantitative real-time PCR was performed in 384-well plates using a 10µl reaction volume containing a 1:5 dilution of cDNA, TaqMan Gene Expression Master Mix, and specific primer-probe sets for the genes of interest and internal controls (all from Applied Biosystems). Amplification was carried out using a ViiA7 Sequence Detection system (Applied Biosystems) under the following cycling conditions: 50 cycles 95°C for 15 seconds and 60°C for 30 seconds. Positive controls were generated from serial dilutions of plasmids containing the amplicons of HIV-1 gag (a kind gift from Rémi Cheynier) (19) or of HIV-2 gag (18). Gene expression levels were normalised to the geometric mean of two housekeeping genes (*GAPDH* and *HPRT*).

### 2.6 Quantification of total cell-associated HIV DNA by droplet digital PCR

Freshly isolated PBMCs were pelleted, dried, and cell lysates prepared with RLT Buffer (Qiagen) and stored at -80°C. DNA was isolated with Quick-DNA/RNA Micropep Plus Kit (Zymo Research) and digested with EcoRI enzyme (10 U/μl, Thermo Fisher Scientific). The ddPCR was performed on the Bio-Rad QX200 AutoDG Digital Droplet PCR system using the ddPCR Supermix for probes (no dUTPs; Bio-Rad Laboratories). The ddPCR was designed to quantify cell-associated HIV-1 or HIV-2 DNA through gag detection with RPP30 gene used to estimate cell amounts. Primers and probes used in ddPCR are listed in **Supplementary Table 3**. The ddPCR reactions consisted of 20μl mixture per well, containing 2,5μl of digested DNA, 10μl supermix for probes (no dUTPS), 10μM of HIV gag probe (Integrated DNA Technologies), 50μM of HIV gag forward and reverse primers (Invitrogen), 1x RPP30 probe and primers (Thermo Fisher Scientific) and DNase/RNase-Free Water (Sigma-Aldrich). The ddPCR reactions were incorporated into droplets using the QX100 Droplet Generator (Bio-Rad). Nucleic acids were amplified with the following cycling conditions: 10min at 95°C, 45 cycles of 30s at 94°C and 58°C for 60s, and a final droplet curve step of 10 min at 98°C using a T100 Thermal Cycler (Bio-Rad Laboratories). Each sample was quantified in triplicate, and replicate wells were merged during analysis to increase accuracy. In parallel, non-template controls were processed and used as a reference for sample amplification. Droplets were read and analysed using Bio-Rad QX200 system and QuantaSoft software (v1.7.4.0917) in ‘absolute quantification’ mode. Only wells containing ≥12,000 droplets were accepted for further analysis. Results are expressed as total cell-associated HIV DNA copies per 10^6^ PBMCs or naïve CD4 T cells.

### 2.7 Statistical analysis

Statistical analysis was performed using Graph-Pad Prism (v9.5.0, GraphPad Software) and R (v4.3.2; R Foundation for Statistical Computing). For R, the ggpubr package (v0.6.0) was used for statistical testing and ggplot2 (v3.5.1) for data visualisation. Kruskal-Wallis was used to compare groups, and for *post-hoc* comparisons, Dunn’s tests were applied using the Holm-Bonferroni correction. For the IL-7 study, Mann-Whitney or Wilcoxon tests were also used as appropriate. Spearman’s rank coefficients were used for correlations, and visualisation of significant correlations produced with the ComplexHeatmap package (v2.18.0). A *p*-value < 0.05 was considered statistically significant.

## 3 Results

### 3.1 Profiling the naïve compartment of people with HIV

**Figure 1:**
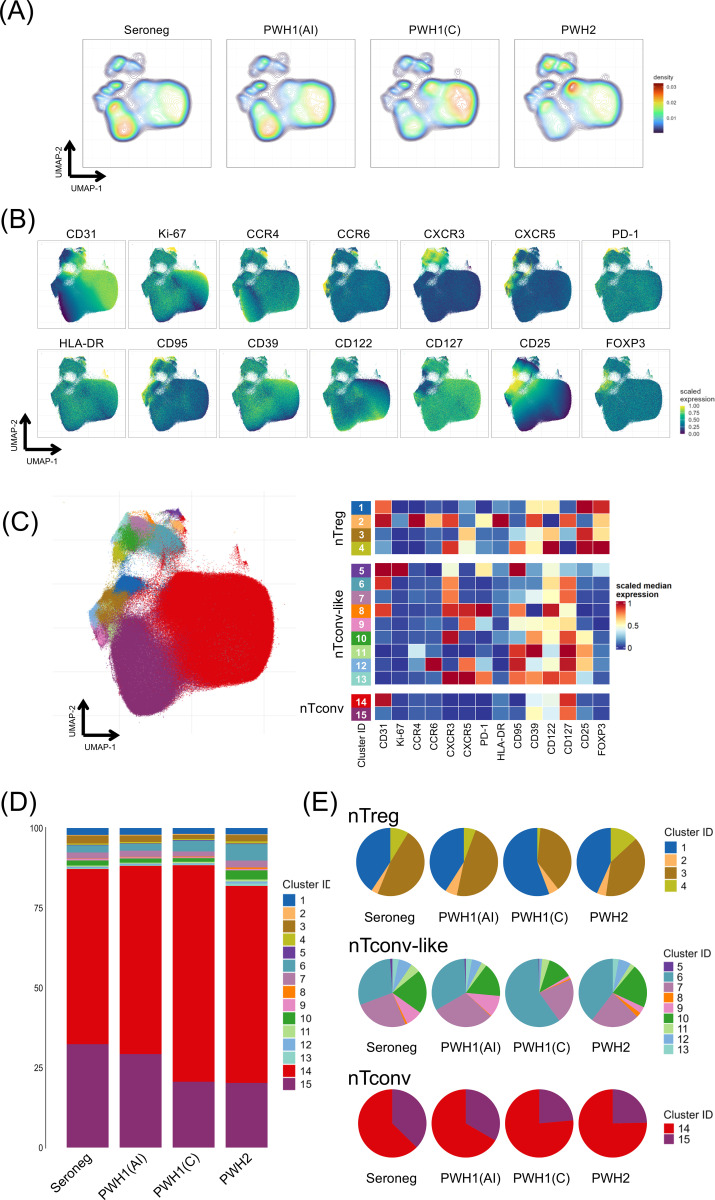
Naïve CD4 T cell heterogeneity in virus-suppressed PWH1 and PWH2. **(A)** UMAP density plots of circulating naïve CD4 T cells from PWH1 that started ART during the acute infection (PWH1(AI), n=16), or during the chronic stage (PWH1(C), n=19), PWH2 with undetectable viremia (PWH2, n=18), and seronegative controls (seroneg, n=21). **(B)** expression of all the markers used to generate the UMAP visualisation of naïve CD4 T cells from all study participants (n=81). **(C)** UMAP displaying the 15 identified clusters in naïve CD4 T cells from the 81 study participants (left); heatmap of median marker expression in each cluster (right) with the 3 main naïve subpopulations manually annotated, namely regulatory T cells (nTregs), conventional cells expressing non-naïve markers (nTconv-like), and the expected conventional cells (nTconv). **(D)** stacked bar plots of the average frequency of each naïve cluster within total naïve CD4 T cells in the 4 groups evaluated. **(E)** pie-charts of the average distribution of the clusters within nTreg (top), nTconv-like (middle), and nTconv (bottom) subpopulations in the 4 cohorts. See also **Supplementary Figures 1** and **2**, supporting materials.

We hypothesised that the type of HIV infection and the duration of plasma viral load exposure impact the naïve CD4 T cell compartment. Therefore, we gathered three cohorts of PWH known to have undetectable viremia for more than 4 years, but with distinct histories of plasma viral load exposure: PWH1(AI), who experienced high HIV-1 viremia for only a few weeks due to ART initiation at initial Fiebig stages of acute HIV-1 infection (n=16) (27, 28); PWH1(C) who experienced plasma HIV-1 load for several years before commencing ART, due to diagnosis during the chronic stage of infection (n=19); and PWH2 who experienced low to undetectable plasma HIV-2 levels throughout the disease course (n=18), even before ART initiation (24). **Table 1** depicts the clinical and epidemiological data from all three cohorts and age-matched seronegative controls (n=21) included in this study. Notably, the proportion of naïve cells within total CD4 T cells was not statistically different between the groups, though the blood counts of naïve CD4 T cells were significantly lower in both PWH1(C) and PWH2 as compared to seronegative controls (**Table 1**). Taking advantage of the potential of spectral flow cytometry, we designed a panel to profile the circulating naïve CD4 T cells immediately after blood collection (see **Supplementary Table 1**, Supporting Materials). In addition to the key naïve markers, we included markers associated with CD4 T cell differentiation and activation, and the host-restriction factor SAMHD1, known to be targeted by the vpx protein of HIV-2 (16, 17). **Figure 1A** shows the density plots of concatenated data from all individuals from each cohort, projected onto the UMAP generated by unsupervised analysis of gated naïve CD4 T cells (see **Supplementary Figure 1A**, Supporting Materials) from all samples. **Figure 1B** shows the expression of the main parameters on the UMAP. We found that the profile of PWH1(AI) was very similar to seronegative controls, whereas PWH1(C) and PWH2 featured distinct imbalances (**Figure 1A**).

Using an unsupervised approach to uncover naïve CD4 T cell heterogeneity, we next performed a clustree analysis using FlowSOM (see **Supplementary Figure 1B**, Supporting Materials), which identified 15 clusters within the naïve CD4 T cell compartment (see **Supplementary Figure 1C**, Supporting Materials), whose projection onto the UMAP is shown in **Figure 1C**. As illustrated in the heatmap (**Figure 1C**), clusters 14 and 15 show no significant expression of markers associated with CD4 T cell differentiation and may therefore be considered the conventional naïve T cell clusters (nTconv). They differed only by the presence (cluster 14) or absence (cluster 15) of CD31 expression. Four clusters were found expressing FOXP3 (clusters 1-4, **Figure 1C**), which were classified as naïve regulatory T cells (nTreg). Nine clusters (clusters 5-13, **Figure 1C**) expressed various differentiation markers in the absence of FOXP3, which were therefore labelled as naïve conventional-like T cells (nTconv-like).

**Figure 2:**
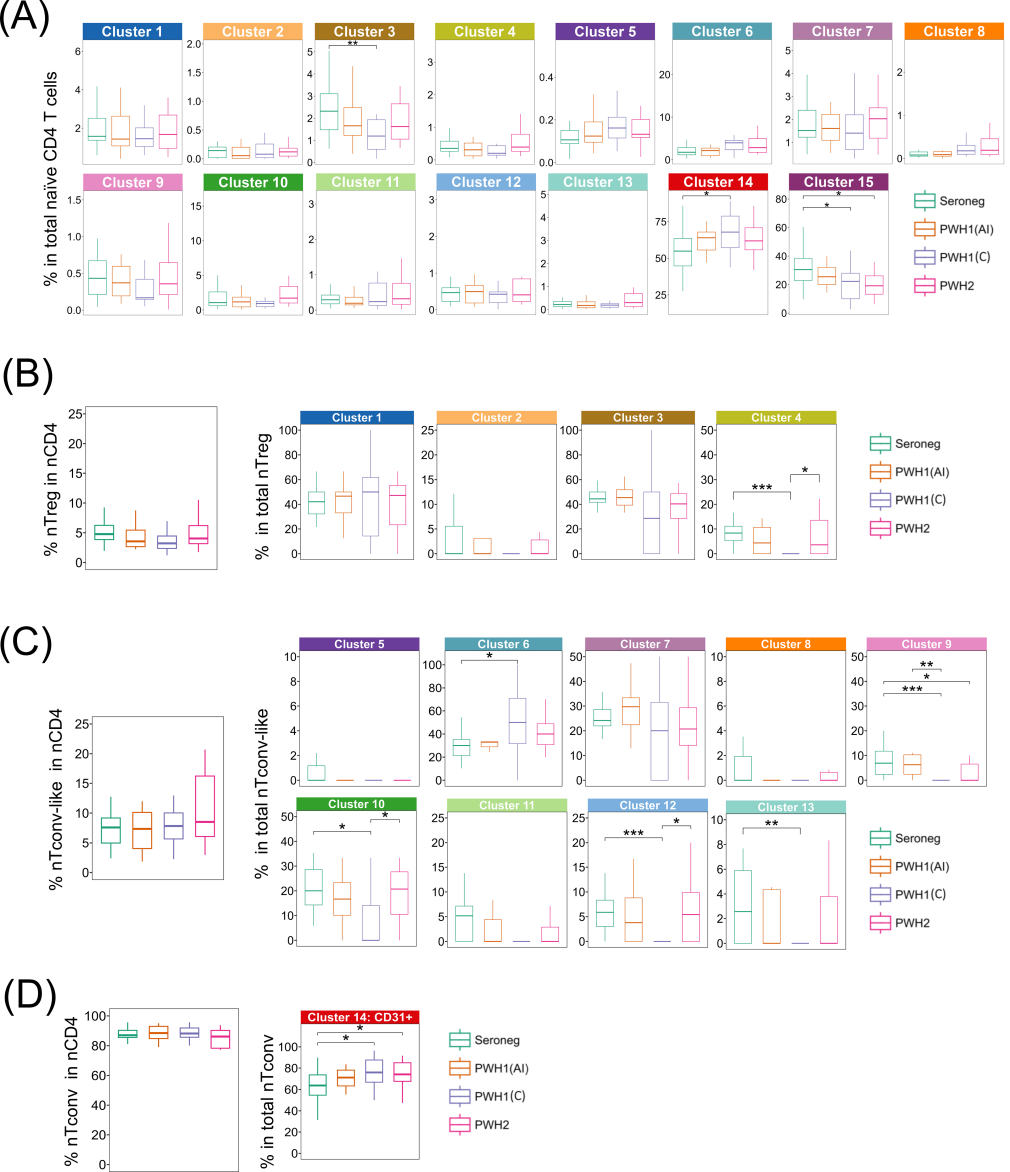
Analysis of the naïve CD4 T cell cluster frequency within total naïve CD4 T cells and main naïve CD4 T cell subpopulations. Comparison of virally suppressed PWH1 grouped according to ART starting in acute or chronic infection (PWH1(AI), n=16; and PWH1(C), n=19), PWH2 with undetectable viremia (PWH2, n=18), and seronegative controls (seroneg, n=21). Boxplots depict: **(A)** the proportion of each cluster within total naïve CD4 T cells; **(B)** the proportion of the merged Treg clusters within total naïve CD4 T cells (left graph) and the relative proportion of each nTreg cluster within total nTregs; **(C)** the proportion of the merged nTconv-like clusters within total naïve CD4 T cells (left graph) and the relative proportion of each nTconv-like cluster within total nTconv-like; **(D)** the proportion of the merged nTconv clusters within total naïve CD4 T cells (left graph) and the relative proportion of CD31+ nTconv cluster within total nTconv. Data represent median and interquartile ranges. Group comparison done with Kruskal-Wallis, and Dunn’s tests using the Holm-Bonferroni correction for *post-hoc* comparisons. Significant *p*-values are shown: *<0.05; **<0.01; ***<0.001.

The mean relative proportion of each cluster within total naïve CD4 T cells was relatively preserved in PWH1(AI) as compared to seronegative, which contrasted with PWH1(C), who featured a significant expansion of cluster 14 (nTconv CD31^+^), and contraction of clusters 3 (nTregs expressing CXCR5, potentially naïve follicular Tregs) and 15 (nTconv CD31^neg^), as shown in **Figure 1D**. Notably, cluster 15 was also significantly contracted in PWH2 (**Figures 1D, E**, **2A**).

These changes became more apparent when the analysis was performed within the three manually annotated populations (**Figures 1E**, **2B–D**).

Within nTregs, we observed a significant contraction of cluster 4 in PWH1(C) compared to both seronegative individuals and PWH2 (**Figure 2B**). This cluster expressed CXCR3, CD95, and high levels of CD122 (the β-chain of the IL-2 receptor), a combination of markers associated with conventional memory stem-cells (8).

Within nTconv-like cells, cluster 6, defined by the expression of CXCR3, CD122 and CD31 in the absence of CD95, was significantly expanded in PWH1(C) (**Figures 1E**, **2C**). In parallel, PWH1(C) showed significant contraction of the clusters 10 (CXCR3+CD39+CD122+CD95^neg^CD31^neg^), 12 (CCR6+CXCR5+CD95+CD122+CD31^neg^), and 13 (CXCR3+CXCR5+PD-1+CD95+CD39+CD122+CD31^neg^), as well as of the cluster 9 (CXCR5+CD39+CD95+CD122+CD31^neg^), which was also contracted in PWH2, albeit less than in PWH1 (**Figures 1E**, **2C**).

Within the nTconv population, both PWH1(C) and PWH2 exhibited significant expansion of the CD31^+^ cluster (**Figures 1E**, **2D**). This finding prompted further investigation of CD31^+^ nTconv cells through manual gating (see **Supplementary Figure 2A**, Supporting Materials), including assessments of CD31 expression levels (see **Supplementary Figure 2B**, Supporting Materials), the proportion of cycling cells (Ki-67^+^; see **Supplementary Figure 2C**, Supporting Materials), and SAMHD-1 expression (see **Supplementary Figure 2D**, Supporting Materials), in both CD31+ and CD31^neg^ nTonv subsets. However, no significant differences were found between the four cohorts.

In summary, PWH1(C) under sustained suppression with effective ART exhibited significant imbalances in the naïve CD4 T cell compartment. Some of these alterations were also observed in PWH2, despite their characteristically low to undetectable viremia throughout the disease course, irrespective of ART. Remarkably, no alterations were found in PWH1(AI), who featured a naïve CD4 T cell profile closely resembling that of seronegative controls.

### 3.2 Determinants of the naïve CD4 T cell imbalances

**Figure 3:**
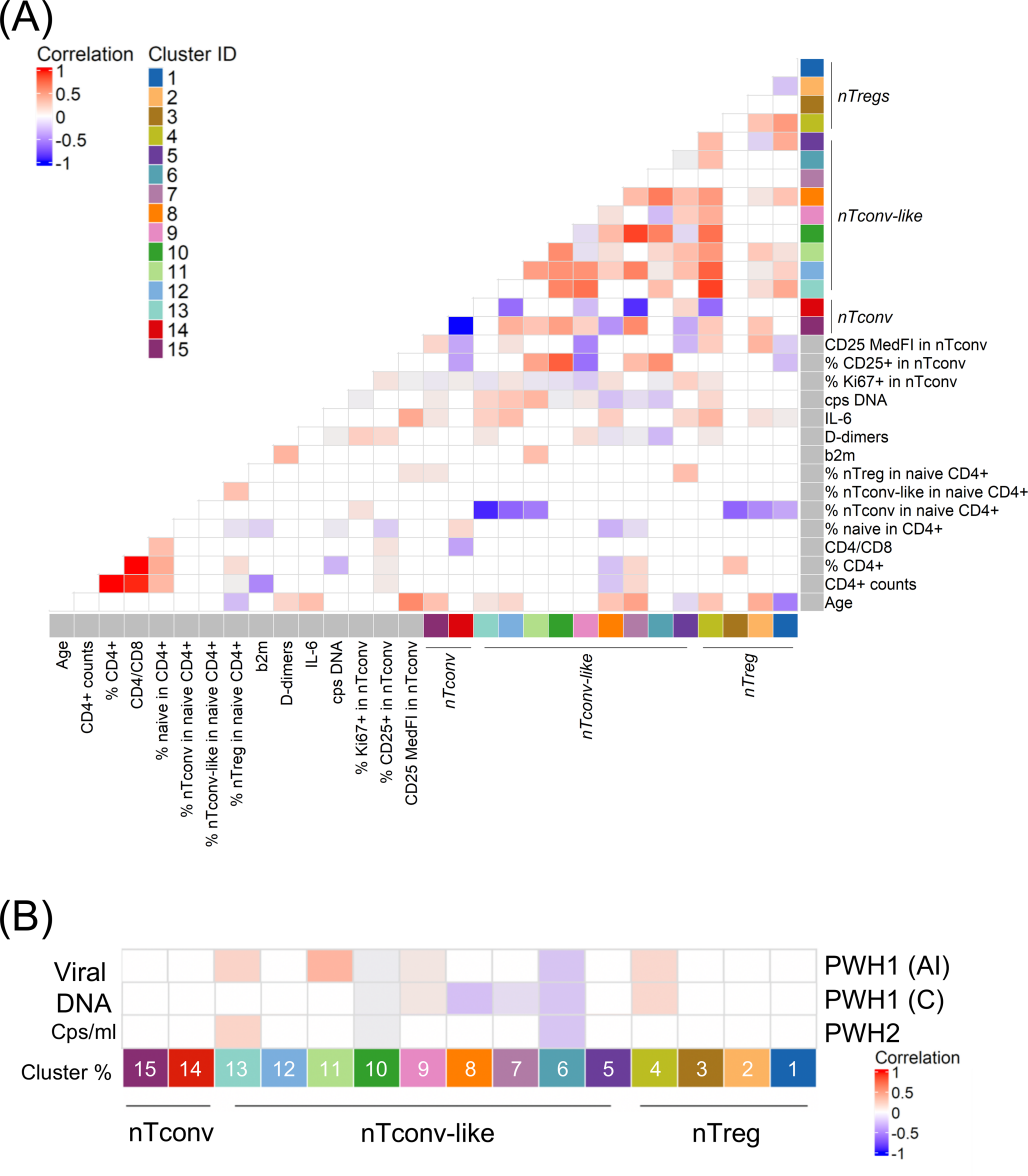
Correlation of naïve CD4 T cell clusters with age, immunological and virological parameters. **(A)** correlogram between the frequencies of the 15 identified clusters within naïve CD4 T cells, the main annotated subpopulations, and selected clinical parameters from **Table 1**; analysis performed in 74 subjects, including all PWH with undetectable viremia (n=53) and seronegative controls (n=21). **(B)** correlation between total cell-associated viral DNA evaluated in PBMCs and the cluster frequency within naïve CD4 T cells in PWH1 that started ART during the acute infection (PWH1(AI), n=16), or during the chronic stage (PWH1(C), n=19), and PWH2 with undetectable viremia (PWH2, n=18). The colour scale refers to Spearman’s rank coefficients. Only significant correlations (*p* < 0.05) are shown. See also **Supplementary Figure 3**, supporting materials.

Next, we investigated possible associations between the observed naïve CD4 T cell imbalances and age, inflammatory and viral parameters. As shown in the correlogram (**Figure 3A**), age was positively associated with the frequency of cluster 15 (CD31^neg^ nTconv), consistent with the known age-related expansion of this subpopulation (4), as well as with the median CD25 expression levels within conventional naïve cells (total nTconv), as previously reported (34). Among the nTconv-like clusters, age was positively correlated with clusters 8, 12, and 13, expressing CXCR5, and cluster 7, which expressed CXCR3 and CD122. Conversely, age was negatively associated with the proliferating cell population represented by cluster 5 (**Figure 3A**). Within the nTreg compartment, age was inversely correlated with cluster 1 (bona-fide CD31+ nTregs) and positively associated with clusters 2 and 4, which exhibit features of activated and stem cell-like nTregs, respectively (**Figure 3A**).

We also identified several associations between cluster frequencies and inflammatory parameters, particularly between serum IL-6 levels and nTconv-like clusters (**Figure 3A**). These associations were more prominent in PWH than in seronegative individuals, as illustrated in the cohort-specific correlograms (see **Supplementary Figure 3**, Supporting Materials).

Some correlations between cluster frequencies were consistent across all participants and within individual cohorts, likely reflecting the variable dependency (**Figure 3A** and see **Supplementary Figure 3**, Supporting Materials). For example, the correlation of cluster 15 (CD31^neg^ nTconv) with several other clusters, while others appeared to be associated with PWH cohorts, such as correlations involving cluster 4 (nTregs expressing memory-stem-cell markers).

Notably, levels of cell-associated viral DNA were not significantly different between PWH1(AI) and PWH1(C) (**Table 1**), consistent with the early establishment of viral reservoirs during acute infection (35). PWH2 exhibited significantly lower levels of cell-associated viral DNA compared to both PWH1 cohorts (**Table 1**). Notwithstanding this, when we assessed whether naïve CD4 T cell imbalances correlated with cell-associated viral DNA, we found a relatively consistent correlation profile across all three PWH cohorts. Specifically, cell-associated viral DNA was negatively associated with the frequency of nTconv-like clusters 6 and 10, both expressing CXCR3 and CD122 (**Figure 3B**).

**Figure 4:**
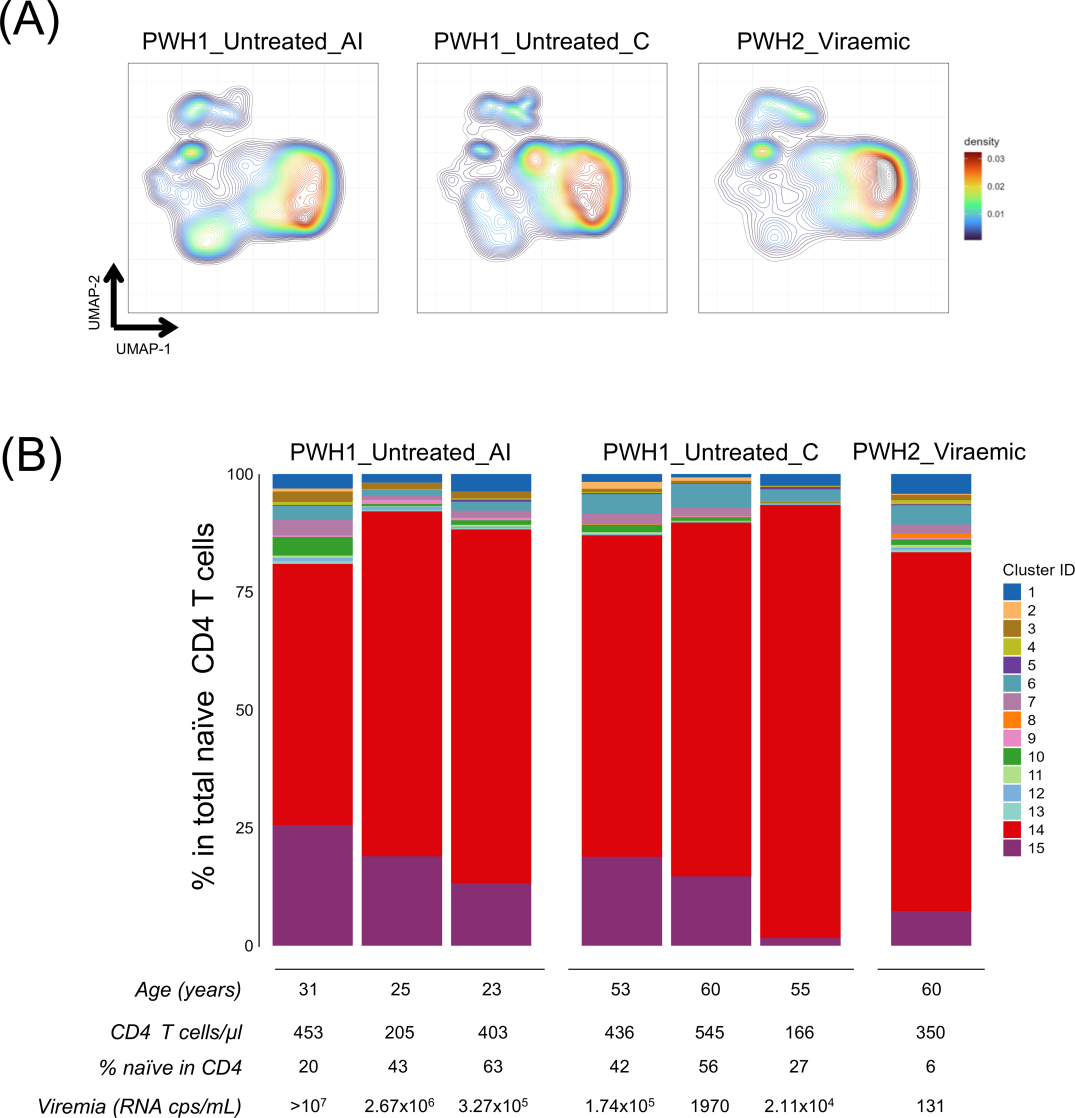
Naïve CD4 T cell heterogeneity in viraemic PWH1 and PWH2. **(A)** density plots of circulating naïve CD4 T cells from viraemic PWH1 recently diagnosed in the acute infection (PWH1_untreated_AI, n=3), and in the chronic stage (PWH1_untreated_C, n=3) before starting ART, and in one PWH2 with detectable viremia (PWH2, n=1). **(B)** stacked bar plots of the cluster distribution within total naïve CD4 T cells from each viraemic subject, with the respective age, CD4 T cell counts, frequency of naïve cells within total CD4 T cells, and plasma viral load depicted below.

In parallel, we profiled the naïve CD4 T cell compartment in seven viraemic PWH and found major perturbations irrespective of disease stage and type of infection (**Figures 4A, B**). Data interpretation was limited by the small number of subjects recruited before the initiation of effective ART, as well as by the considerable interindividual heterogeneity. Interestingly, cluster 14 (CD31+ nTconv) appeared more expanded in individuals at more advanced stages of disease, despite their older age (**Figure 4B**).

Thus, although our data support an impact of the cell-associated viral burden in naïve CD4 T cells, other contributors, like the length of viremia exposure, are necessary to explain the lack of imbalances documented in PWH1 treated during the acute infection.

### 3.3 Naïve CD4 T cell ability to respond to IL-7

IL-7 is the main homeostatic cytokine for human naïve CD4 T cells and a key contributor to the maintenance of the CD31+ subset (6). Therefore, we next asked whether intrinsic properties of naïve CD4 T cells in PWH modulate their responsiveness to IL-7, reasoning that both the type of HIV infection and the presence of plasma viral load might have an impact. To address this possibility, we relied on a study using sort-purified naïve CD4 T cells from age-matched untreated PWH1, PWH2, and seronegative controls (**Table 2**), which was conducted in our laboratory before the use of ART was made universal.

All PWH were ART-naïve and featured relatively preserved CD4 T cell counts to avoid potentially confounding immunopathologic factors associated with advanced HIV infection and to ensure the required cell yields. In addition, we included a group of PWH1 elite controllers (EC) to enable comparison of ART-naïve PWH1 with PWH2 with undetectable viremia (24, 29, 30). Despite the differences in plasma viral load, all PWH featured similarly elevated levels of T cell activation (**Table 2**), consistent with our previous observations that T cell activation correlates with CD4 T cell depletion in both PWH1 and PWH2 (24).

**Figure 5:**
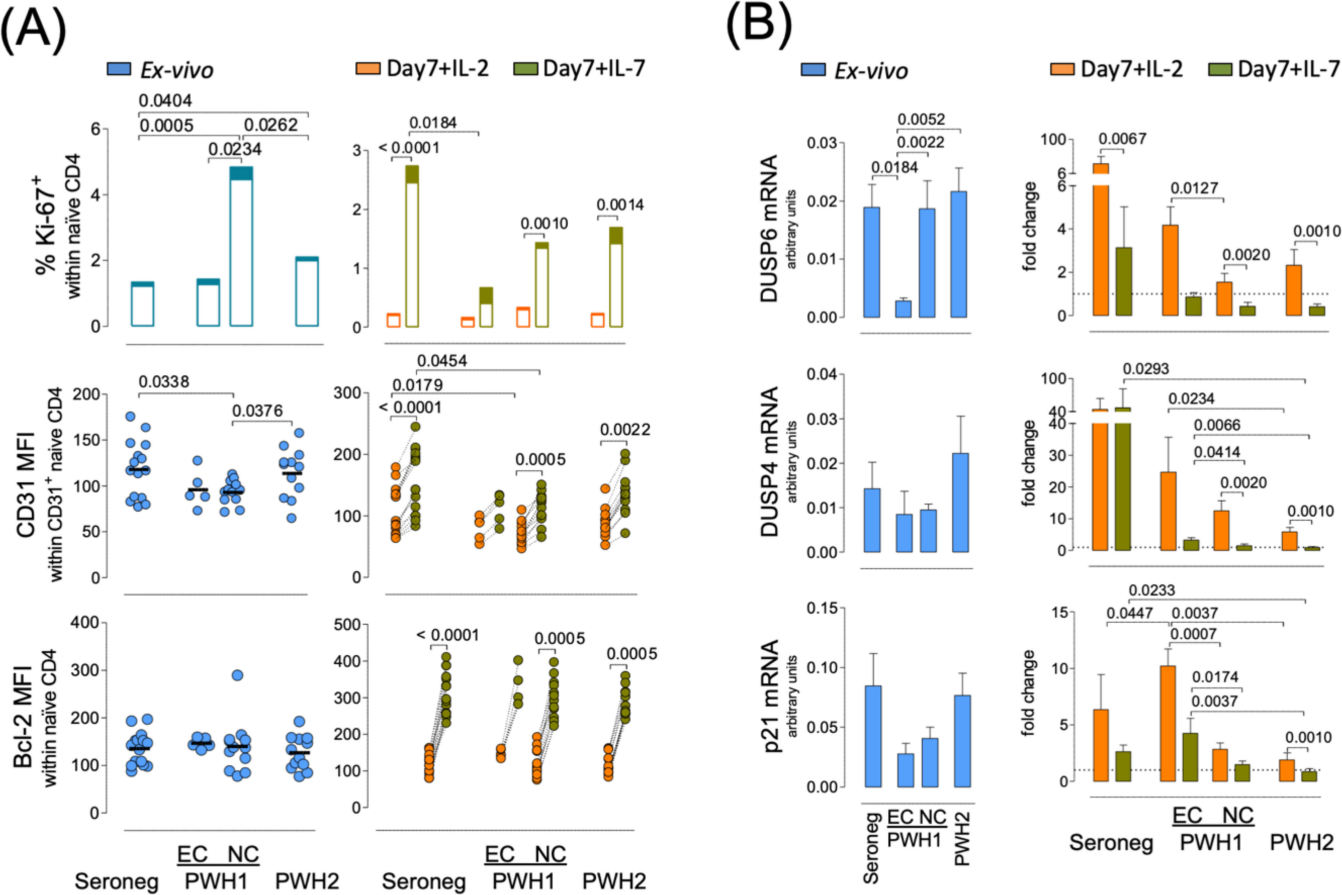
Naïve CD4 T cell ability to respond to *in vitro* IL-7 stimulation. Total naïve CD4 T cells from PWH1 elite-controllers (EC) and non-controllers (NC), PWH2 and seronegative (seroneg) cohorts were sort-purified and analysed regarding their phenotype *ex vivo* (left graphs; blue) and upon 7-day culture with IL-7 or IL-2 (right graphs; green and orange, respectively). **(A)** frequency of cycling (Ki-67+) cells and the relative contribution of CD31+ (open bars) and CD31^neg^ (filled bars) to the pool of cycling naïve CD4 T cells (top graphs); mean fluorescence intensity (MFI) of CD31 within CD31+ naïve CD4 T cells (middle graphs); and Bcl-2 MFI (bottom graphs); each dot or pair of connected dots represents one individual, bars represent median. **(B)** transcriptional levels of *DUSP6* (top graphs); *DUSP4* (middle graphs); and *p21* (bottom graphs); *ex vivo* levels were normalised to those of housekeeping genes (*HPRT* and *GAPDH*) and results expressed as 2^(-ΔCt)^; levels in cultured cells were normalised to those of housekeeping genes and the corresponding basal levels (*ex vivo*), and results expressed as 2^(-ΔΔCt)^; bars represent mean ± SEM. Inter- and intra-cohort data were compared using Mann-Whitney or Wilcoxon tests, respectively. *p*-values <0.05 were considered significant and are shown.

Although both PWH2 and PWH1-EC experienced prolonged infection, they relatively preserved naïve CD4 T cells (**Table 2**). Notably, the overall proportion of CD31+ cells within the naïve CD4 T cell pool was comparable across all cohorts (see **Supplementary Figure 4**, Supporting Materials), despite a reduction in the mean fluorescence intensity of CD31 expression within the CD31+ subset in PWH1 (**Figure 5A**). Moreover, there were no significant differences between the levels of expression of the IL-7Rα (CD127, MFI levels: seronegative 51 ± 8; PWH1-EC 34 ± 4; PWH1-NC 35 ± 4; PWH2 33 ± 4). PWH1 non-controllers (PWH1-NC) exhibited significantly elevated *ex vivo* frequencies of cycling naïve CD4 T cells (Ki-67+), in both CD31+ and CD31^neg^ subsets, relative to seronegative, PWH2 and PWH1-EC (**Figure 5A**). This finding suggests a potential role for ongoing HIV-1 antigenemia in driving peripheral proliferation of naïve CD4 T cells. While PWH1-EC showed no increase in the frequency of cycling naïve T cells, a significant increase was found in PWH2 (**Figure 5A**), indicating that distinct mechanisms may underlie the maintenance of the naïve T cell compartment across PWH subgroups. Notably, the expression levels of the anti-apoptotic protein Bcl-2 in naïve CD4 T cells were similar across all cohorts (**Figure 5A**).

To investigate the contribution of IL-7 to naïve CD4 T cell homeostasis in PWH, sort-purified naïve CD4 T cells were cultured for 7-days in the presence of IL-7 or IL-2 as a control. The latter is included to circumvent the high rate of cell death observed in isolated naïve T cells cultured without cytokine support and was previously shown not to impact the maintenance of the naïve phenotype (6). Cell death/survival were estimated by the levels of pro-survival, Bcl-2, and pro-apoptotic, Fas (CD95), factors, as well as by the expression of the marker of early apoptosis Annexin 5.

All cohorts maintained the proportion of CD31+ cells in culture (see **Supplementary Figure 4A**, Supporting Materials) and upregulated CD31 expression in response to IL-7 stimulation (**Figure 5A**). IL-7 stimulation induced comparable upregulation of the anti-apoptotic protein Bcl-2 across all cohorts (**Figure 5A**). Furthermore, IL-7 stimulation, relative to IL-2, led to a reduction in the frequency of apoptotic cells (as measured by Annexin 5 levels) despite concurrent upregulation of the pro-apoptotic receptor Fas in all evaluated cohorts, as previously described in response to IL-7 (36) (see **Supplementary Figure 4B**, Supporting Materials). However, the proliferative responses to IL-7 were reduced in PWH, with PWH1-EC showing a statistically significant reduction compared to seronegative (**Figure 5A**). Collectively, these data indicate that naïve CD4 T cells from PWH exhibit an impaired proliferative response to IL-7, with the defect being most pronounced and statistically significant in PWH1-EC despite the low numbers.

These observations led us to explore molecular regulators associated with naïve T cell quiescence and homeostatic signals, specifically the transcription factors KLF2 and Foxp1, known to modulate naïve quiescence and differentiation into memory (37, 38), the cell-cycling regulator p21 that has been implicated in elite-controller status (39), and the dual-specificity phosphatases DUSP4 and DUSP6, which may modulate TCR thresholds for activation and responses to IL-7 (40, 41). To this end, total RNA was extracted from sort-purified naïve CD4 T cells *ex vivo* and after 7-day culture in the presence of IL-7 or IL-2. Transcript levels were quantified via real-time qPCR. In seronegative individuals, *ex vivo KLF2* mRNA levels negatively correlated with the percentage of cycling naïve CD4 T cells (r=-0.6214, p=0.0134), a relationship not observed in PWH1. Despite this, *ex vivo KLF2* expression was not significantly different among cohorts and declined similarly in culture, regardless of cytokine stimulation (see **Supplementary Figure 4C**, Supporting Materials). *Foxp1* was significantly overexpressed in PWH1-EC as compared to seronegative (see **Supplementary Figure 4C**, Supporting Materials), potentially contributing to the lack of *ex vivo* proliferation observed in this group (**Figure 5A**). However, a consistent reduction in *Foxp1* expression was observed upon culture in all PWH, independent of viremia, in contrast to the stability seen in seronegative individuals (see **Supplementary Figure 4C**, Supporting Materials). PWH1-EC exhibited the lowest *ex vivo p21* mRNA levels but showed marked induction of *p21* expression following IL-7 or IL-2 stimulation (**Figure 5B**). This aligns with previous reports suggesting that PWH1-EC may resist HIV infection via *p21* upregulation (39), and could also partially explain their diminished IL-7-induced proliferative responses (**Figure 5A**). In contrast, PWH2 exhibited a significantly impaired ability to upregulate *p21* in response to IL-7 compared to seronegative individuals (**Figure 5B**), underscoring mechanistic differences between PWH2 and PWH1-EC despite their shared ability to control viral replication without ART. Lastly, regarding *DUSP4* and *DUSP6*, PWH1-EC showed a markedly reduced *ex vivo* expression of *DUSP6*, significantly lower than in all other cohorts (**Figure 5B**). While seronegative individuals demonstrated robust induction of both *DUSP4* and *DUSP6* in response to both IL-2 and IL-7, this response was notably blunted in all PWH cohorts (**Figure 5B**).

**Figure 6:**
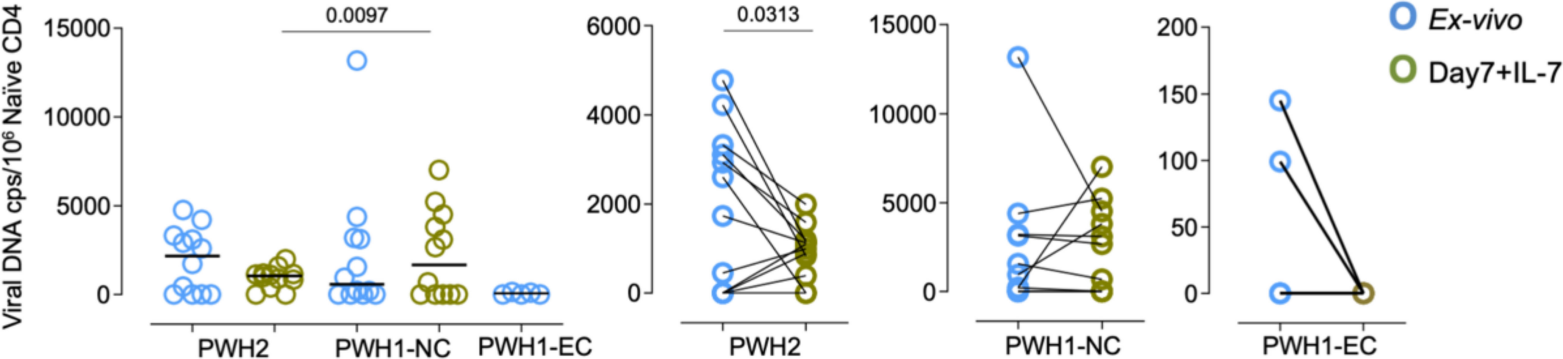
Impact of IL-7 stimulation on viral DNA in naïve CD4 T cells. Total naïve CD4 T cells from PWH1 elite-controllers (EC), PWH1 non-controllers (NC), and PWH2 cohorts were sort-purified and analysed regarding the amount of cell-associated viral DNA *ex vivo* and upon 7-day culture with IL-7. Total cell-associated viral DNA, quantified by ddPCR, before (blue) and after 7-day culture (green); bars are mean ± SEM; each dot or pair of connected dots represents one individual. Mann-Whitney test was used for inter-cohort comparisons (left graph) and Wilcoxon test for intra-cohort comparisons (other graphs). *p*-values<0.05 were considered significant and are shown.

To estimate the possible impact of infected cells we quantified the cell-associated viral DNA in sort-purified naïve CD4 T cells and no significant differences were found between PWH2 and PWH1-NC before culture (**Figure 6**), with viral DNA detectable in 8/12 subjects in each group. In contrast, PWH1-EC had undetectable (3/5) or very low (2/5) cell-associated viral DNA *ex vivo*, which became undetectable in all individuals following 7-day culture with IL-7 (**Figure 6**). Interestingly, upon IL-7 stimulation, the cell-associated viral load was maintained or increased in PWH1-NC, whilst it decreased in PWH2 (**Figure 6**). This is interesting because of the known role of IL-7 in promoting viral replication (42) and the recent evidence of higher cytopathogenicity of HIV-2 upon *in vitro* infection of primary CD4 T cells potentially leading to the elimination of these cells (43, 44).

Overall, these findings suggest that the reduced proliferative responses to IL-7 observed in PWH1-NC, PWH1-EC, and PWH2 may be attributed to distinct intrinsic properties of their naïve CD4 T cells. Impaired upregulation of mRNA transcripts associated with cellular quiescence may render naïve CD4 T cells from PWH more susceptible to low-level TCR stimulation, thereby fuelling T cell activation and the consequent disease progression. Conversely, naïve CD4 T cells from PWH-EC displayed robust induction of transcripts, particularly *p21*, in response to IL-7 stimulation, which may help sustain a state of cellular quiescence and prevent excessive transition of naïve cells into the memory-effector compartment, thus curbing the replenishment of the latently infected CD4 T cell reservoir.

## 4 Discussion

Early antiretroviral treatment during the acute HIV-1 infection preserved the composition of the naïve CD4 T cell compartment, while sustained imbalances were observed in both PWH1(C) and PWH2 who started ART during the chronic stage, irrespective of the CD4 T cell nadir. Most studies comparing early and late ART in PWH1 have mainly focused on the impact on viral reservoirs (45), without including a detailed multiparameter naïve phenotyping beyond the classical markers (CD45RA, CCR7, CD27/28, CD95) used to reveal the preservation of naïve CD4 counts. Our study confirmed the marked heterogeneity of naïve CD4 T cells and showed a similar relative frequency of naïve CD4 T cell clusters in early-treated PWH1 and seronegatives, which agreed with the results obtained by a previous study reporting a transcriptional analysis at the single-cell level (25).

Moreover, distinct patterns of imbalance were identified in late-treated PWH1(C) and PWH2, with potential functional implications, namely the relative expansion of CXCR3^bright^ cells in PWH1(C) and of cells with a full TSCM phenotype (CD95^+^CXCR3^+^CD122^+^CD31^+^) in PWH2, in line with a previous report defining TSCM based only on the expression of CD95 (46). The enrichment in CXCR3^bright^ cells may underscore a poising of naïve CD4 T cells from PWH1(C) for TH1 differentiation and IFNγ production with possible impact on the inflammatory state (23). In addition to the Tconv alterations, our data support a distinct impact on Tregs, with significant underrepresentation in PWH1(C) of cells with a follicular phenotype (TFR) compared to seronegatives, and of TSCM-like naïve Tregs compared to both seronegative and PWH2. Persistent inflammation has been shown to modulate Tregs, inducing distinct Treg populations defined by functional states (47–49). Since both PWH1(C) and PWH2 maintained systemic inflammation under ART, it would be relevant to disentangle which inflammatory determinants contribute to these distinct phenotypes and the impact of these imbalances on clinical outcomes of persistent inflammation. Additionally, the virus itself may impact naïve cell activation and differentiation, either directly (16, 50) or indirectly, for instance, through dendritic cells (51–54).

Interestingly, both late-treated PWH1(C) and PWH2 showed a relative expansion of CD31+ naïve CD4 T cells. This population, which includes the RTEs, is known to decline with ageing and immune senescence (4). It is plausible that the enrichment in CD31+ naïve cells results from a long-lasting homeostatic response mediated by IL-7, which preserve the diversity of the TCR repertoire better than self-peptide MHC/TCR stimulation (42, 55, 56). We have shown that IL-7 upregulates CD31 expression and promotes preferential proliferation of the CD31+ subset (1, 6). Since IL-7 also promotes HIV replication (42, 55, 56), we compared here the *in vitro* responses of purified naïve CD4 T cells from untreated PWH1 and PWH2 to IL-7, to better understand the relative impacts on viral production and homeostatic mechanisms. Although both PWH2 and PWH1 elite controllers had undetectable viremia in the absence of ART, their IL-7 responses were markedly distinct. Elite controllers had a unique profile of DUSP6 expression *ex vivo* and p21 upregulation upon IL-7 stimulation, underscoring a preponderant role of host genetics in this response, through mechanisms that remain largely unclear (39, 57, 58). We did not find other studies that examined IL-7 responses in isolated naïve CD4 T cells from elite controllers, and the small size of our cohort of these extremely rare individuals limits the strength of our conclusions. Nevertheless, it is plausible that the poor proliferative ability has a protective effect on viral dynamics and disease progression (12). IL-7 responses mediated by PI3K (6) were markedly impaired in PWH1 non-controllers and to a lesser extent in PWH2, potentially reflecting thymic dysfunction, since RTEs are the primary responders (41). Notably, we observed comparable levels of proviral DNA in naïve CD4 T cells from PWH1 non-controllers and PWH2; however, while HIV-1 levels remained stable during IL-7 culture, HIV-2 levels declined significantly. Our findings suggest that IL-7 promoted HIV-2 replication, which led to the death of the infected cells. This hypothesis is supported by studies showing that HIV-2 exhibits reduced fitness but greater cytopathogenicity than HIV-1 in primary human cells (43, 44), which may impact the naïve CD4 T cell homeostasis (59, 60).

Although it is increasingly difficult to study fresh cells from untreated PWH, our findings, together with our profiling of the naïve CD4 T cell compartment under ART, stress the importance of this largely overlooked subset. Other single-cell approaches, including transcriptomics and metabolomics, should further uncover naïve cell states in PWH1 and PWH2 to identify targets to modulate cell renewal and activation, impacting disease outcomes and viral reservoirs (12, 25, 50). The emerging methodologies that combine DNA and RNA sequencing are also expected to clarify the contribution of each subset to the viral reservoir dynamics.

In conclusion, imbalances in naïve CD4 T cell heterogeneity persist in both PWH1(C) and PWH2 under ART, warranting further exploration to identify targets for purging viral reservoirs and achieving complete immune reconstitution, thereby resolving chronic inflammation and immune senescence.

## Data Availability

The raw data supporting the conclusions of this article will be made available by the authors, without undue reservation.
