# Supplementary material for: Shaping the founders: naïve CD4 T cell heterogeneity in people with HIV-1 or HIV-2
Source: Front Immunol. 2026 May 11;17:1810186. doi: 10.3389/fimmu.2026.1810186 (PMC13199175; doi:10.3389/fimmu.2026.1810186)
Supplement: Supplementary file 1 [file DataSheet1.pdf]

Supplementary Material

1 Supplementary Data

Supporting Tables

Supplementary Table 1. Antibodies used in Spectral Flow Cytometry panel

Supplementary Table 2. Antibodies used to evaluate the in vitro response to IL-7 by Flow Cytometry

Supplementary Table 3. Primers/Probes used to quantify cell-associated viral DNA

Supporting Figures

Supplementary Figure 1. Naïve CD4 T cell gating strategy and clustering analysis**.**

Supplementary Figure 2. Analysis of CD31+ naïve conventional CD4 T cells in virus-suppressed PWH1 and PWH2.

Supplementary Figure 3. Correlogram of naïve CD4 T cell subpopulations with age and relevant immunological parameters in virus-suppressed PWH and seronegative controls.

Supplementary Figure 4. Analysis of the ability of naïve CD4 T cells from PWH to respond to IL-7.

2 Supplementary Tables

2.1 Supplementary Table 1

Supplementary Table 1. Antibodies used in Spectral Flow Cytometry panel

Marker	Clone	Fluorochrome	Source
FOXP3	206D	BV421	BioLegend
CD8	RPA-T8	eFluor 450	eBiosciences
CCR6	11A9	BV480	BD Biosciences
PD-1	EH12.2H7	BV605	BioLegend
HLA-DR	L243	BV650	BioLegend
CCR7	G043H7	BV711	BioLegend
CCR4	1G1	BV750	BD Biosciences
CD31	WM59	BV785	BioLegend
SAMHD-1	l19-18	FITC (conjugated in-house)	Merck
CD45	HI30	Alexa Fluor 532	Invitrogen
CD4	RPA-T4	PerCP-Cy5.5	Invitrogen
CD39	eBioA1	PerCP-eFluor710	Invitrogen
CD95	DX2	PE	Invitrogen
CXCR5	J252D4	PE-Dazzle594	BioLegend
CD25	BC96	PE-Cy5	BioLegend
CXCR3	G025H7	PE-Cy7	BioLegend
CD122	TU27	APC	BioLegend
Ki-67	B56	Alexa Fluor 647	BD Biosciences
CD127	A019D5	Alexa Fluor 700	BioLegend
CD45RO	UCHL1	APC-H7	BD Biosciences
CD3	SK7	APC-Fire810	BioLegend

2.2 Supplementary Table 2

Supplementary Table 2. Antibodies used to evaluate the in vitro response to IL-7 by Flow Cytometry

Marker	Clone	Fluorochrome	Source
CD4	RPA-T4	FITC	eBiosciences
CD45RA	HI100	FITC	eBiosciences
HLA-DR	L243	FITC	BD Biosciences
Bcl-2	124	FITC	Dako
CD38	HB7	PE	eBiosciences
CD95	DX2	PE	eBiosciences
CD127	40131	PE	R&D Systems
Ki-67	B56	PE	BD Biosciences
CD3	OKT3	PerCP	eBiosciences
CD4	SK3	PerCP	eBiosciences
CD45RA	HI100	PerCP	eBiosciences
CD27	O323	PE-Cy7	eBiosciences
CD8	RPA-T8	APC	eBiosciences
CD31	WM59	APC	eBiosciences
CD3	SK7	APC-Cy7	BD Biosciences
CD4	RPA-T4	APC-Cy7	BD Biosciences
CD8	SK1	APC-Cy7	BD Biosciences

2.3 Supplementary Table 3

Supplementary Table 3. Primers/Probes used to quantify cell-associated viral DNA

Primer/Probe	Gene	Sequence
Forward	HIV-1 gag	CGAGAGCGTCAGTATTAAGC
Reverse	HIV-1 gag	AGCTCCCTGCTTGCCCATAC
Probe	HIV-1 gag	5'-FAM-CCCTGGCCTTAACCGAATT-MGB-3'
Forward	HIV-2 gag	CGCGAGAAACTCCGTCTTG
Reverse	HIV-2 gag	GCTGCCCACACAATATGTTTTA
Probe	HIV-2 gag	5'-FAM-CCGGGCCGTAACCT-MGB-3'
Forward	RPP30	GCTGTGTTTGCTCTCTTGATTT
Reverse	RPP30	GGTCTGTCCATGGCATCTTAT
Probe	RPP30	5'-HEX-AATGTCTGTGACTGGGTTCTGGCT-MGB-3'

3 Supplementary Figures

3.1 Supplementary Figure 1

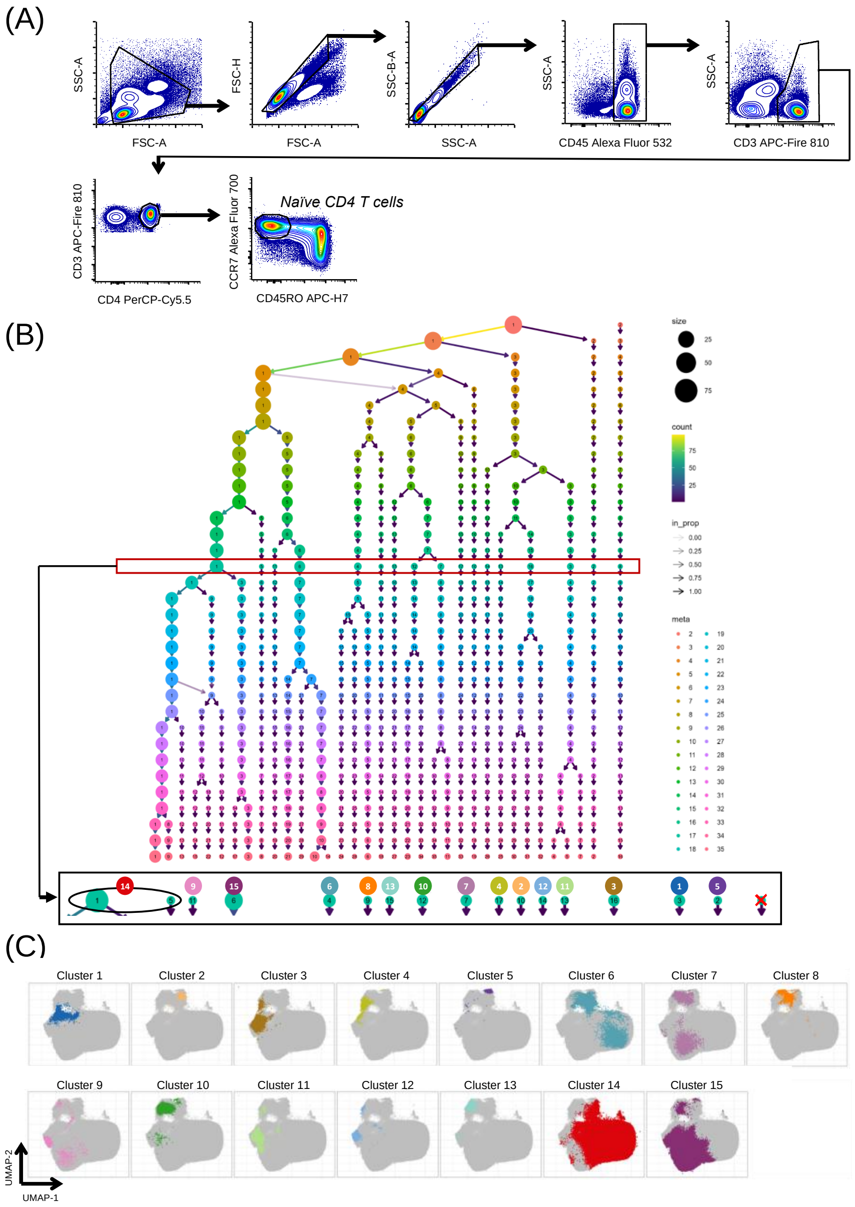

Supplementary Figure 1. Naïve CD4 T cell gating strategy and clustering analysis. (**A**) Illustrative manual gating strategy to obtain the naïve CD4 T cells, which were exported as fcs files (FCS Express) and imported into R for unsupervised analysis. (**B**) Clustree analysis of naïve CD4 T cells from all donors using FlowSOM. Resolution meta17 (highlighted with a red box) was selected based on tree stabilisation and used as a starting point for the final cluster identification; the bottom box highlights the identified 15 clusters with their respective colours and numbers. (**C**) UMAP plots of naïve CD4 T cells from all donors showing the projection of each cluster in their assigned colour. Refer to Figures 1 and 2.

3.2 Supplementary Figure 2

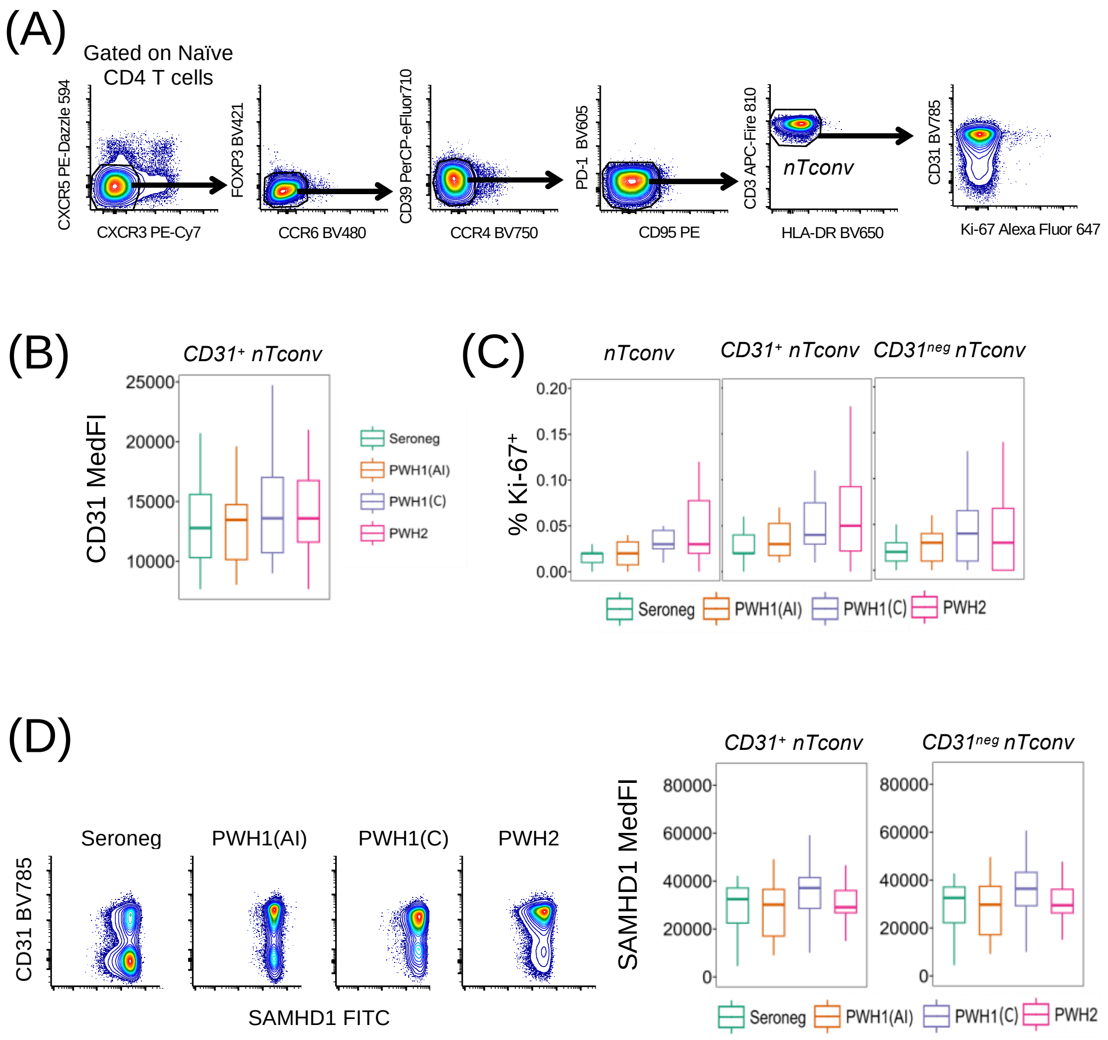

Supplementary Figure 2. Analysis of CD31+ naïve conventional CD4 T cells in virus-suppressed PWH1 and PWH2. (**A**) Illustrative manual analysis of the nTconv in a representative subject (PWH2, 34y, 704 CD4 T cells/µl, 26% naïve within CD4 T cells). (**B**) Boxplot comparing the median fluorescence intensity (MedFI) of CD31 within CD31+ nTconv in the 4 cohorts, namely PWH1 that started ART during the acute infection (PWH1(AI), n=16), or during the chronic stage (PWH1(C), n=19), PWH2 with undetectable viremia (PWH2, n=18), and seronegative controls (Seroneg, n=21). (**C**) Comparison of the frequency of proliferating cells (Ki67+) within total nTconv, CD31+ nTconv, and $CD31^{neg}$ nTconv in the 4 cohorts. (**D**) Illustrative examples of the manual flow cytometry analysis of intracellular SAMHD1 staining and surface CD31 in gated nTconv from representative subjects of the 4 cohorts (Seroneg, 59y, 1206 CD4 T cells/µl, 33% naïve within CD4 T cells; PWH1(AI), 41y, 680 CD4 T cells/µl, 40% naïve within CD4 T cells; PWH1(C), 60y, 669 CD4 T cells/µl, 10% naïve within CD4 T cells; PWH2, 70y, 1115 CD4 T cells/µl, 57% naïve within CD4 T cells) and boxplots of SAMHD1 MedFI within gated CD31+ nTconv (left) and CD31neg nTconv (right) of subjects from the 4 groups (14 PWH1(AI); 19 PWH1(C); 14 PWH2 and 17 Seroneg). Data represent median and interquartile ranges. Group comparison done with Kruskal-Wallis, and Dunn's tests using the Holm-Bonferroni correction for post-hoc comparisons. No significant *p*-values (<0.05) were found.

3.3 Supplementary Figure 3

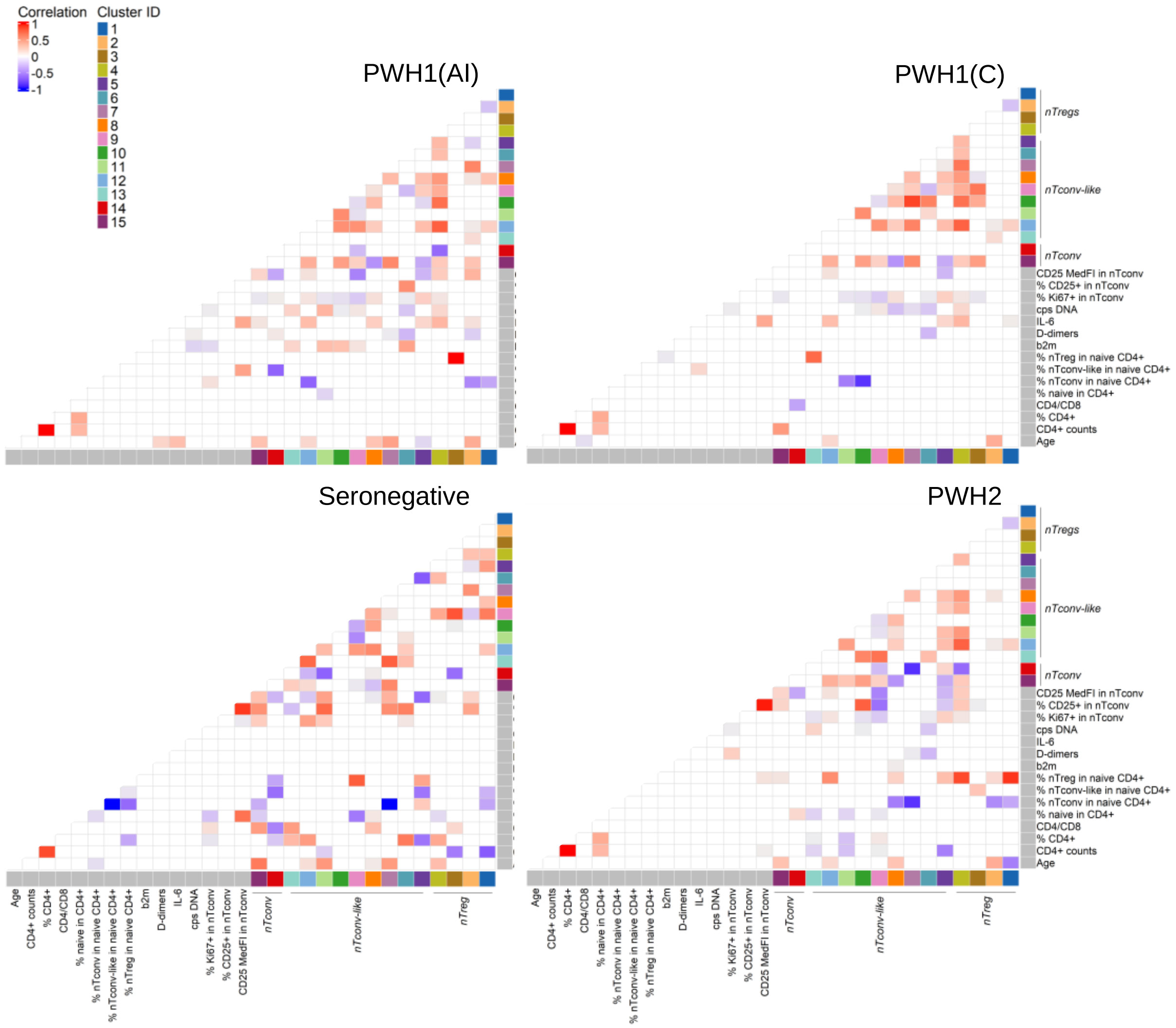

Supplementary Figure 3. Correlogram of naïve CD4 T cell subpopulations with age and relevant immunological parameters in virus-suppressed PWH and seronegative controls. Analysis performed in PWH1 who started ART during the acute infection (PWH1(AI), n=16, top left), or during the chronic stage (PWH1(C), n=19, top right), PWH2 with undetectable viremia (PWH2, n=18, bottom right), and seronegative controls (Seronegative, n=21, bottom left). The colour scale refers to Spearman's rank coefficients between the frequencies of the 15 identified clusters, the main annotated subpopulations within naïve CD4 T cells, and selected clinical parameters from Table 1. Only significant correlations ($p < 0.05$) are shown. Refer to Figure 3.

3.4 Supplementary Figure 4

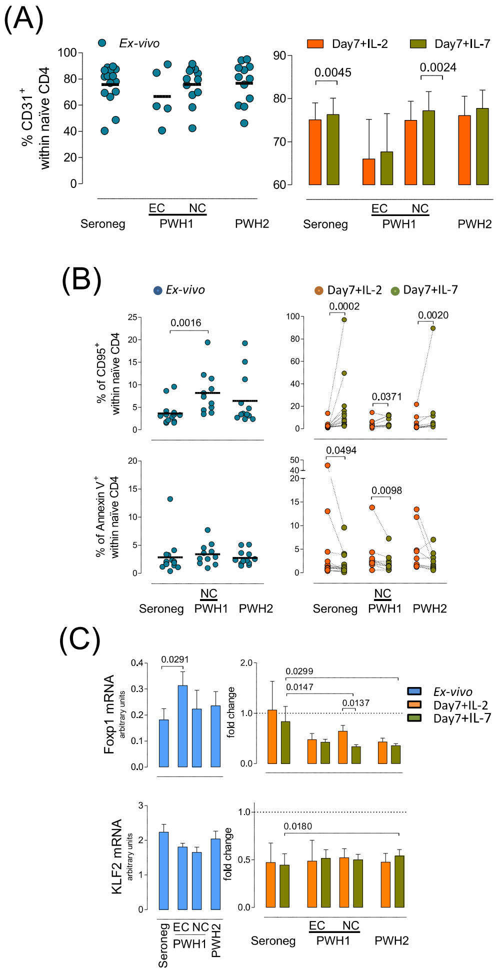

Supplementary Figure 4. Analysis of the ability of naïve CD4 T cells from PWH to respond to IL-7. Total naïve CD4 T cells from PWH1 elite-controllers (EC) and non-controllers (NC), PWH2 and seronegative (Seroneg) cohorts were purified, and analysed by flow cytometry ex vivo (left graphs; blue), and after 7-day culture with either IL-7 or IL-2 (right graphs; green and orange, respectively). (**A**) Frequency of CD31+ cells. (**B**) Proportion of cells expressing CD95 (top graphs) and Annexin V+ (bottom graphs). Each dot or pair of connected dots represents one individual, bars represent median. (**C**) *FOXP1* and *KLF2* transcript levels (upper and lower graphs, respectively); ex vivo levels were normalised to those of housekeeping genes (*HPRT* and *GAPDH*) and results expressed as $2^{(-\Delta Ct)}$; levels in cultured cells were normalised to those of housekeeping genes and the corresponding basal levels (ex vivo), and results expressed as $2^{(-\Delta\Delta Ct)}$; bars represent mean±SEM. Inter- and intra-cohort data were compared using Mann-Whitney or Wilcoxon tests, respectively. *p*-values <0.05 were considered significant and are shown. Refer to Figure 5.
